# Gene editing innovations and their applications in cardiomyopathy research

**DOI:** 10.1242/dmm.050088

**Published:** 2023-05-24

**Authors:** Eirini Kyriakopoulou, Thomas Monnikhof, Eva van Rooij

**Affiliations:** ^1^ Hubrecht Institute, Royal Netherlands Academy of Arts and Sciences (KNAW) and University Medical Center, 3584CT Utrecht, The Netherlands; ^2^ Department of Cardiology, University Medical Center Utrecht, 3584CX Utrecht, The Netherlands

**Keywords:** Base editing, Genetic cardiomyopathy, Prime editing, Therapy

## Abstract

Cardiomyopathies are among the major triggers of heart failure, but their clinical and genetic complexity have hampered our understanding of these disorders and delayed the development of effective treatments. Alongside the recent identification of multiple cardiomyopathy-associated genetic variants, advances in genome editing are providing new opportunities for cardiac disease modeling and therapeutic intervention, both *in vitro* and *in vivo*. Two recent innovations in this field, prime and base editors, have improved editing precision and efficiency, and are opening up new possibilities for gene editing of postmitotic tissues, such as the heart. Here, we review recent advances in prime and base editors, the methods to optimize their delivery and targeting efficiency, their strengths and limitations, and the challenges that remain to be addressed to improve the application of these tools to the heart and their translation to the clinic.

## Teaser

**Summary:** Despite remaining challenges, recent advances in genome editing, particularly with prime and base editors, offer exciting new possibilities for modeling and treating genetic cardiomyopathies.

## Introduction

Cardiomyopathies refer to structural and/or functional abnormalities affecting the myocardium that arise in the absence of definite underlying disease able to cause the myocardial pathology (Elliott et al., 2007). Patients diagnosed with cardiomyopathies often present with impaired cardiac function and an increased risk of heart failure, a severe clinical syndrome characterized by an imbalance between cardiac output and oxygen demand. Heart failure is currently considered an emerging pandemic (Groenewegen et al., 2020), which also imposes enormous healthcare costs through morbidity, high rates of hospitalization, premature mortality and lost productivity (Cook et al., 2014).

Cardiomyopathies are categorized in different ways (see Box 1) and are characterized by high levels of clinical and genetic complexity, making their early diagnosis and treatment a challenge (Teekakirikul et al., 2013). Dilated cardiomyopathy (DCM) is the most prevalent cardiomyopathy worldwide and a leading cause of heart transplantation. A reliable population-based epidemiological study about DCM prevalence and incidence is currently missing. However, recent estimations describe an approximate prevalence from 1:250 to 1:500 up to 1:2500 people globally (Hershberger et al., 2021). Hypertrophic cardiomyopathy (HCM) cases have been identified in 122 countries of the world with a combined population of 6.3 billion people, which accounts for 88% of the world population. Estimates of HCM prevalence originate disproportionally from developed countries with mature medical systems and suggest an occurrence of 1:500 in the general population based on clinical expression of the disease phenotype. However, this is considered to be an underestimation given the limited geographical coverage, variable clinical presentation and low penetrance of the disease (Maron Barry et al., 2018). Arrhythmogenic cardiomyopathy (ACM) has an estimated prevalence varying from 1:1000 to 1:5000, with the discordance often attributed to misdiagnoses. Even though ACM is known to occur throughout the world, it has historically been linked to Northeastern Italy, where ACM-related sudden cardiac death incidents were more common than in other countries. Large cohorts sharing founder mutations are found in The Netherlands and in North America, while other forms of non-syndromic ACM occur in Asia and Africa. In the United States, ACM is more prevalent in white people, whereas individuals with Asian and African ancestry are also represented in well-characterized patient cohorts (Corrado et al., 2017). Restrictive cardiomyopathy (RCM) is the rarest of the major cardiomyopathies, mostly caused by acquired and not genetic factors. Genetic RCM is lethal, but accounts for less than 5% of all cardiomyopathy cases (Cimiotti et al., 2021).

**Box 1. History of cardiomyopathy classification**

The first classification system for cardiomyopathies originated from the World Health Organization (WHO) in 1980 (McKenna et al., 2017; No author listed, 1980). The proposed system, merely based on the observed structural and hemodynamic phenotypes, divided cardiomyopathies into dilated (DCM), hypertrophic (HCM) and restrictive (RCM) idiopathic cardiomyopathies, meaning that they constituted ‘heart muscle diseases of unknown cause’. The first evidence of genetic predisposition associated with cardiomyopathy came in the early 1990s, when cardiomyopathies were no longer considered idiopathic for many patients (Richardson et al., 1996). Back in 1966, the WHO introduced arrhythmogenic cardiomyopathy (ACM), and the new ‘unclassified cardiomyopathies’ category was created. Forty years later, the American Heart Association (AHA) categorized cardiomyopathies as primary and secondary (Maron et al., 2006); primary cardiomyopathies were defined as the ones predominantly confined to heart muscle and were further classified as ‘genetic’, ‘mixed’ and ‘acquired’. In contrast, the term ‘secondary’ implied that the cardiomyopathy is part of a multi-systemic disease such as sarcoidosis or amyloidosis. Although the American classification system was mainly based on etiology, the European classification, published in 2008 by the European Society of Cardiology, classified cardiomyopathy based on functional and structural phenotypes, which was in line with the WHO recommendation (Elliott et al., 2007). Each of the phenotypes described, namely DCM, HCM, ACM, RCM and unclassified cardiomyopathy (including left ventricular non-compaction), was further subclassified into familial/non-familial forms. Notably, the European classification system acknowledged, for the first time, the potential involvement of genetics in disease progression and detection within families. With more recent insights into the complex genetics of cardiomyopathy development and progression, the MOGE(S) classification system has been proposed (Arbustini et al., 2013) and endorsed by the World Heart Federation. The MOGE(S) system describes the morphofunctional phenotype (M), organ(s) involvement (O), genetic inheritance pattern (G), etiological annotation (E) including genetic defect or underlying disease/substrate, and the disease state (S). It thus integrates phenotypic description with genetic information, helping to better distinguish between overlapping phenotypes and genetic syndromes. An example of MOGE(S) classification of a DCM patient: they presented with an atrioventricular block (M) without any organ involvement (O), described as a familial disease with autosomal-dominant inheritance pattern (G). Etiologies (E) included the presence of a pathogenic *LMNA* variant and the detection of a parvovirus B19 (B19V) in the heart. Last, the disease stage is classified as level D (according to the AHA scaling system) and class IV (according to the New York Heart Association scaling system): M_D(AVB)_ O_(H)_ G_G(AD)_ E_G-LMNA[pArg190Trp]+V-B19V_ S_D-IV_.

Despite holding promise for facilitating cardiomyopathy prognosis, the MOGE(S) classification system was not integrated into routine clinical practice due to the complexity and expertise needed to properly apply this classification. Thus, the need for a concise and comprehensive classification system to better represent the complexity and heterogeneity of cardiomyopathies is still unmet.

Individual genetic variants associated with cardiomyopathy are often incompletely penetrant and show variable expressivity, and can cause mild or severe forms of the disease (McNally et al., 2015a). In addition, secondary and/or additional mutations can contribute to the development and progression of cardiomyopathy, challenging the established ‘single-gene disorder’ concept of this disease (Girolami et al., 2010; Golbus et al., 2014). Recent exome- and genome-sequencing studies have identified almost 100 genes that are associated with the onset of cardiomyopathy, substantially improving our understanding of the underlying genetic mechanisms leading to different types of the disease. As an example of this, ∼75% of HCM cases are attributed to mutations in the thick filament genes myosin-binding protein C (*MYBPC3*) and β-myosin heavy chain (*MYH7*) (Ingles et al., 2019). MYH7 and MYBPC3 are both essential components of the sarcomere (see Glossary, Box 2), with MYH7 providing the force for muscle contraction and MYBPC3 playing a regulatory role in muscle contraction and stabilizing the thick filament. Mutations in the encoding genes can disrupt the structure of the sarcomere, leading to abnormal thickening of the heart muscle and other complications associated with HCM (Lopes et al., 2013). DCM, which exhibits greater genetic heterogeneity than HCM, is commonly associated with mutations in the titin (*TTN*) gene (Herman et al., 2012; Horvat et al., 2019). Titin serves as a scaffold within the sarcomere, connecting the thick and thin filaments and playing a critical role in determining the elasticity of the muscle fiber. The elastic properties of titin enable it to store and release energy during the cardiac cycle, allowing the heart to pump blood efficiently. Mutations in *TTN* can lead to a decrease in titin expression or to the formation of abnormal titin proteins, which can affect the elasticity of the heart muscle and lead to its dilation and subsequent heart failure (Herman et al., 2012). Arrhythmogenic cardiomyopathy (ACM) is associated with plakophilin 2 (*PKP2*) gene mutations; *PKP2* encodes a component of the desmosome (Box 2) that connects adjacent cardiomyocytes, thus maintaining the structural integrity of the tissue (Gerull et al., 2004; Bourfiss et al., 2022). Mutations in *PKP2* can lead to abnormal desmosome function and structure, subsequently causing cardiomyocyte detachment, increased apoptosis, inflammation and fibrosis. These changes can ultimately lead to the replacement of cardiac muscle with fibrofatty tissue, disrupting the normal electrical conduction of the heart and leading to severe ventricular arrhythmias. In addition to *PKP2* mutations, mutations in other desmosome-related genes have also been associated with ACM, highlighting the important role that these structures play in the development and progression of the disease. Lastly, mutations in the troponin I3 (*TNNI3*) gene can lead to RCM by disrupting the regulation of cardiac muscle contraction and relaxation. Mutations in *TNNI3* can affect the function of the encoded protein troponin I, leading to the stiffness of the heart muscle that characterizes RCM (Mogensen et al., 2015).

**Box 2. Glossary**

**Activity window:** the target nucleotides within the protospacer in which the base editor is active, of which the first nucleotide at the 5′ end is considered number 1 and number 20 is the first nucleotide proximal of the protospacer adjacent motif (PAM).

**Desmosome:** a type of cell junction that anchors adjacent cells together in tissues that experience mechanical stress, such as skin and heart muscle.

**Engineered pegRNAs (epegs):** prime editing guide RNAs (pegRNAs) with 3′ RNA motif after the extension that prevents against degradation by endonucleases.

**Exon skipping:** a form of RNA splicing used to cause cells to ‘skip’ over faulty or misaligned sections (exons) of genetic code, leading to a truncated, but still functional, protein despite the genetic mutation.

**Homology-directed repair (HDR):** a process by which a cell repairs a DNA double-stranded break (DSB) in the presence of a repair template (e.g. sister chromosome or exogenous DNA strand). Only occurs during the S and G2 phases of the cell cycle.

**Non-homologous end joining (NHEJ):** an error-prone DNA repair mechanism that can join two broken DNA ends together without requiring a repair template. May occur throughout cell division.

**PEmax editors:** prime editors with a codon-optimized reverse transcriptase and an enhanced nuclear localization signal.

**Postmitotic:** a condition in which a cell is no longer capable of dividing.

**R-loop:** a three-stranded nucleic acid structure that forms when a single-stranded RNA molecule hybridizes with the DNA template strand during transcription.

**Sarcomere:** the basic structural unit of a muscle fiber, consisting of thick and thin filaments that slide past each other to cause muscle contraction.

**Split-intein system:** a process whereby two protein fragments bind to form a catalytically competent enzyme, then catalyze their own excision and the ligation of their flanking sequences.

**Transgenic models:** experimental *in vitro* and *in vivo* models that have their genome altered to include foreign DNA for the purpose of studying the function of a gene of interest.

**Tropism:** the ability of a virus or other pathogen to infect specific types of cells or tissues in the body.

Cardiomyopathy is also a major clinical feature of select neuromuscular disorders, particularly the X-linked recessive Duchenne muscular dystrophy (DMD) and Becker muscular dystrophy (BMD), with a prevalence of ∼1:5000 (Verhaert et al., 2011). The dystrophin (*DMD*) gene is mutated in both syndromes and encodes a cytoskeletal protein that maintains the structural integrity of muscle fibers during contraction-relaxation cycles (Ervasti, 2007). DMD is characterized by loss-of-function mutations in *DMD* that lead to a complete absence of dystrophin, while in BMD, the DMD protein is truncated and partly functional, resulting in a less severe condition (Strehle and Straub, 2015; Hoffman et al., 1989). In both disorders, DCM is reported as the leading cause of death (Hoffman et al., 1989). Of note, there is a higher mortality among patients diagnosed with DMD cardiomyopathy than among those with other dilated cardiomyopathies (McNally et al., 2015b).

Numerous studies have enriched our knowledge of the pathophysiology and clinical presentation of the most prevalent cardiomyopathies (Kimura, 2016). Nevertheless, despite recent progress, the current available therapeutic approaches for treating genetic cardiomyopathies focus on treating the symptoms of the disease rather than on targeting its underlying genetic causes. In recent years, the cardiovascular field has greatly benefited from genome editing technologies, particularly CRISPR-related technologies, which have enabled the development of clinically relevant *in vivo* and *in vitro* disease models to investigate the underlying genetic and molecular mechanisms of cardiomyopathy (van Kampen and van Rooij, 2019). In this Review, we provide an overview of the contributions that genome editing tools are making to cardiac disease modeling and targeting. We discuss their strengths and limitations, the techniques being developed to improve their targeting and efficiency, and the challenges that remain to be overcome to apply these tools more effectively to cardiac cells and tissue. We begin the Review by providing a brief introduction to each main genome editing technology and then discuss their applications in cardiac research. For a more detailed description of these tools, please see a recent review by Richardson et al. (2023).

## CRISPR-Cas9 technology

**Fig. 1.**
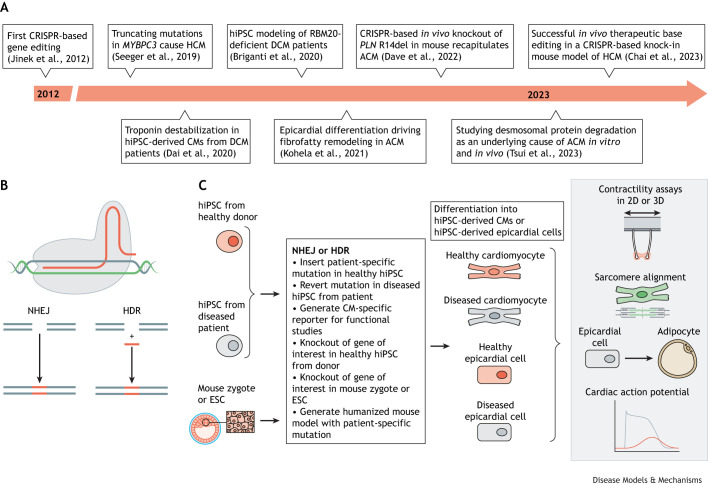
**Impactful reports and schematic of CRISPR-based gene editing to generate disease models recapitulating the genetic cardiomyopathies studied *in vitro*.** (A) Timeline of keystone research reports. (B) CRISPR-Cas proteins are guided to the genomic target site by guide RNAs to generate DSBs induced by the RuvC and HNH nuclease domains. The DSB typically occurs three base pairs upstream of the PAM. Two DNA repair systems are exploited to complete editing: NHEJ and HDR. NHEJ is often used to generate gene-specific knockout models. HDR can generate patient-specific or transgenic models, because of the possibility to insert a donor template. (C) HDR is often used to induce or correct specific mutations, for example for reverting patient-specific hiPSCs to isogenic controls using a donor template (left), so differentiated hiPSC-derived CMs can be phenotypically compared *in vitro* (right). Alternatively, patient-specific mutations can be knocked-in to hiPSCs from healthy donors. NHEJ is utilized to study the effect of a gene knockout or knockout of a pathogenic variant in hiPSC-derived CMs. After successful generation of the hiPSCs with or without the desired mutation, the cells are differentiated into CMs or other cardiac cell types for *in vitro* studies*.* Besides molecular assays, current functional studies investigate differences in contractility, sarcomere alignment, electrophysiology and cell differentiation, which has also been done by generation of CM-specific reporter lines. ACM, arrhythmogenic cardiomyopathy; CM, cardiomyocyte; DCM, dilated cardiomyopathy; DSB, double-stranded break; ESC, embryonic stem cell; HDR, homology-directed repair; HCM, hypertrophic cardiomyopathy; hiPSC, human induced pluripotent stem cell; NHEJ, non-homologous end joining; PAM, protospacer adjacent motif; 2D, two dimensions; 3D, three dimensions.

CRISPR-Cas gene editing tools consist of a Cas nuclease, which is directed to specific regions of the genome by a single-guide RNA (sgRNA) (Jinek et al., 2012). Once the sgRNA-Cas9 nuclease complex has bound its target DNA and has recognized the protospacer adjacent motif (PAM), the double-stranded DNA (dsDNA) unwinds, and the two active domains HNH and RuvC are activated and induce a double-stranded break (DSB) 3 bp proximal to the PAM (Jiang et al., 2016, 2015; Szczelkun et al., 2014; Garneau et al., 2010; Jinek et al., 2014). DSB induction activates two main DNA repair pathways: non-homologous end joining (NHEJ; Box 2) and homology-directed repair (HDR; Box 2) (Fig. 1A) (Chapman et al., 2012; Yeh et al., 2019). NHEJ often results in small insertions or deletions (indels) and is mostly used to generate gene-specific knockout models. HDR-mediated DSB repair is widely used to generate precise edits that are the basis of patient-specific or transgenic models (Box 2) (Fig. 1B) (Maruyama et al., 2015; Chu et al., 2015; Richardson et al., 2016; van Kampen and van Rooij, 2019).

## CRISPR-Cas9 applications in cardiac research

Classical CRISPR-Cas9 technology has been used to engineer isogenic lines of human induced pluripotent stem cells (hiPSCs) to investigate the association of genotype to disease phenotype in a precise, robust and reproducible way (Fig. 1C) (Liu and Olson, 2022). This technology has thus allowed the *in vitro* and *in vivo* study of many monogenic diseases of high or incomplete penetrance (Nguyen et al., 2020; van Kampen and van Rooij, 2019). For example, patient-specific hiPSC-derived cardiomyocytes and microtissues harboring a mutation in *PKP2* were shown to display impaired desmosomal stability compared to isogenic controls generated using CRISPR-Cas9. These findings were further validated in a knock-in mouse model recapitulating the human mutation that was generated via CRISPR-mediated gene editing (Tsui et al., 2023). In addition, hiPSC-derived epicardial cells harboring the same mutation spontaneously differentiated to fibroblast or fat cells *in vitro.* This differentiation appeared to be due the desmosomal disruption and could be stimulated by knocking down desmosomal genes in control hiPSC-derived epicardial cells (Kohela et al., 2021). These findings significantly enrich our understanding of the underlying molecular mechanisms leading to the onset of ACM in patients harboring *PKP2* mutations. In addition, Francesca Stillitano and co-workers recently described a humanized mouse model expressing a heterozygous phospholamban (*PLN*) mutation mimicking human patients (Dave et al., 2022). This mutation leads to deletion of Arg14 (R14del) and has been linked to lethal arrhythmias and ventricular dilation in patients. The mutant mice presented with traits that recapitulated the human condition, such as biventricular dilation and increased stroke volume at 12 weeks of age. Importantly, the researchers were able to improve cardiac function in the mutant mice by performing CRISPR-Cas9-mediated *in vivo* genome editing combined with adeno-associated virus (AAV)9 delivery to inactivate the mutant allele. This study provided the first preclinical evidence that CRISPR-mediated disruption of the *PLN* R14del allele can improve cardiac function in a humanized mouse model and could therefore be valuable for treating the arrhythmogenic phenotype in human carriers (Dave et al., 2022).

Genome editing of hiPSCs has contributed a wealth of additional understanding. For example, Lars Steinmetz's group used a two-dimensional *in vitro* model of DCM to show that CRISPR-based knock-in of patient-specific mutations in *RBM20* can lead to splicing defects and impaired contraction in hiPSC-derived cardiomyocytes compared to isogenic controls (Briganti et al., 2020). In addition, patient-specific hiPSC-derived cardiomyocytes carrying *TNNT2* and *MYBPC3* mutations showed impaired contractile function compared to isogenic controls generated using CRISPR-Cas9-mediated rescue (Dai et al., 2020; Seeger et al., 2019), and hiPSC-derived engineered heart muscles (EHMs) harboring a patient-specific mutation in *TNNT2* displayed sarcomeric dysregulation and impaired contractile force compared to isogenic controls (Dai et al., 2020). The same study also demonstrated that EHMs from patient-specific hiPSC-derived cardiomyocytes carrying a mutation in *TNNT2* recapitulated DCM by showing sarcomeric dysregulation and impaired contractile force (Dai et al., 2020). Patient-specific hiPSC-cardiomyocytes harboring a mutation in *MYBPC3* and grown in two-dimensional and three-dimensional cultures showed upregulation of pathways associated with hypertrophy, and especially aberrant calcium signaling, compared to isogenic controls yielded by CRISPR-mediated repair (Seeger et al., 2019). Besides studying genetic cardiomyopathies, CRISPR has been used to generate gene-specific reporter lines to study hiPSC-derived-cardiomyocyte-specific properties, such as sarcomere disarray, contractility and calcium handling (Maria Cherian et al., 2023; Ribeiro et al., 2020; Sharma et al., 2018; Toepfer et al., 2020; Lin et al., 2018).

CRISPR-Cas9 editing has been a successful tool in generating cell lines and mouse models to study genetic cardiomyopathies. However, its translational capacity is limited by the DSB lesions that can cause undesired biological effects, such as cell toxicity, cell death, mutation and transformation to a carcinogenic state, which can alter the disease phenotype (Cox et al., 2015). Furthermore, HDR-mediated genome editing is restricted to the S/G2 phase, which consequently lowers or abolishes its efficacy on slowly or non-dividing cells and makes ‘classic CRISPR’ less applicable as a potential tool for treatment of cardiomyocyte-specific disease in which single-nucleotide variants should be corrected (Orthwein et al., 2015). Base editing and prime editing are considered safer, more efficient and precise technologies for the generation of disease-associated models, especially in postmitotic (Box 2) systems, as they do not rely on DSB induction (Cox et al., 2015). In the next sections, we focus on the latest applications of base editors and prime editors in cardiac disease modeling and treatment.

## Base editing technology

**Fig. 2.**
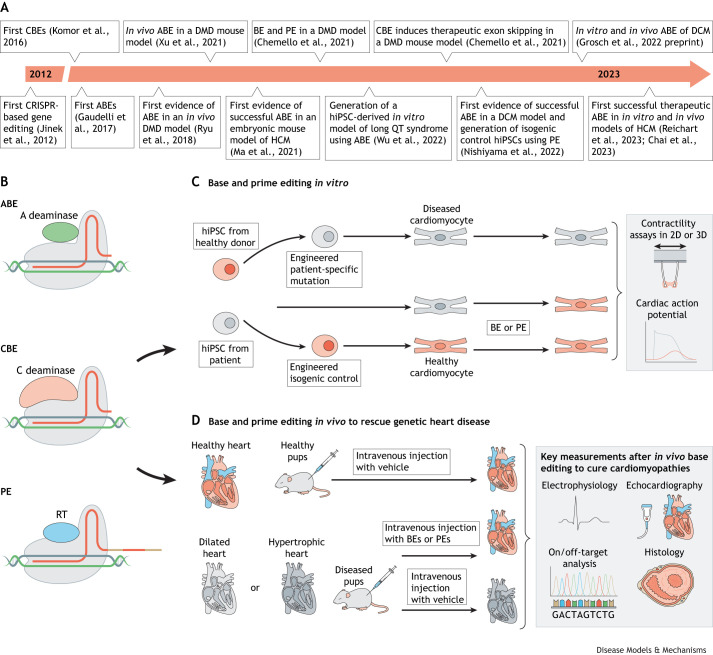
**Timeline of breakthrough *in vivo* studies and schematic of CRISPR-based base and prime editing to rescue and generate disease models recapitulating genetic cardiomyopathies *in vitro* and *in vivo*.** (A) Timeline of keystone research reports. (B) ABEs and CBEs consist of a Cas nickase fused to an adenine or cytidine deaminase, respectively (top, middle), to precisely install C-G to T-A and A-T to G-C mutations. Nicking of the non-deaminated strand triggers DNA repair to introduce the desired mutation. PEs consist of a Cas nickase fused to an RT (bottom). PEs are guided to the genomic target site by engineered pegRNAs containing the spacer, scaffold and RTT, and an RNA motif at the 3′ terminus to enhance stability and prevent degradation. The RTT consists of a PBS (beige), desired edit (red) and a region of homology to genomic DNA (beige). Hybridization of the PBS with the free 3′ end of the genomic DNA primes reverse transcription of the new DNA flap, which is successfully incorporated after DNA repair. (C) Base and prime editing can be used to generate cardiomyopathy-related hiPSCs or to revert mutations in patient-derived hiPSCs (left). After successful generation of cell lines with the mutation(s) of interest, hiPSCs are differentiated to cardiac cell types and used for functional studies or rescue experiments using further base and prime editing. Edited cells are subsequently phenotypically compared with isogenic controls at a molecular and functional level, such as by evaluating contractility and electrophysiological properties (right). (D) Base and prime editing can also be used to generate *in vivo* models that recapitulate genetic cardiomyopathies. In addition, rescue studies to prevent or cure the development of cardiomyopathies are performed *in vivo* to facilitate the translation of gene therapy in cardiomyopathies to the clinic. A, adenine; ABE, adenine base editor; BE, base editor; C, cytidine; CBE, cytosine base editor; DCM, dilated cardiomyopathy; DMD, Duchenne muscular dystrophy; HCM, hypertrophic cardiomyopathy; hiPSC, human induced pluripotent stem cell; PBS, primer-binding sequence; PE, prime editor; pegRNA, prime editing guide RNA; RT, reverse transcriptase; RTT, reverse transcriptase template; 2D, two dimensions; 3D, three dimensions.

Base editing introduces point mutations with precision without DSB induction (Komor et al., 2016). The two main classes of base editors (BEs), the cytosine base editors (CBEs) and the adenine base editors (ABEs), can convert a C-G base pair to T-A, and an A-T to G-C, respectively (Fig. 2A) (Rees and Liu, 2018). Hence, CBEs and ABEs can restore all four transition mutations (C-to-T, A-to-G, T-to-C, G-to-A), representing ∼30% of all annotated human pathogenic variants in the ClinVar database (Porto et al., 2020).

BEs fuse a ssDNA cytidine or adenine deaminase to a Cas9 nickase that encompasses an inactivated RuvC domain (Anzalone et al., 2020). Upon hybridization of the sgRNA to the target of interest, the nucleotides in the R-loop (Box 2) become accessible for deamination if positioned in the correct ‘activity window’ (Box 2). A productive interaction between a C deaminase with its substrate can convert a C-G to T-A, while an A deaminase converts an A-T to G-C (Komor et al., 2016; Gaudelli et al., 2017).

Next-generation ABEs and CBEs are engineered with narrower or broader editing windows, a more active deaminase, or to recognize various PAM sequences to expand the scope of genomic sites of interest that can be targeted in *in vitro* and *in vivo* models of heart disease (Fig. 2B,C) (Rees et al., 2017; Rees and Liu, 2018; Huang et al., 2019a,b, 2021; Tekel et al., 2021; Nishiyama et al., 2022; Lebek et al., 2023; Chai et al., 2023). ABEmax and BE4max are newer versions of ABEs and CBEs, respectively, that present with a higher nuclear localization and a codon-optimized deaminase to increase activity (Koblan et al., 2018).

Although base editing is the most efficient tool to induce or restore the four transition mutations, this genome editing technology is currently accompanied by limitations, of which targetability and delivery are the most prominent (Huang et al., 2021). BEs are catalytically active within a certain window of the spacer, which requires a suitable PAM at the genomic site of interest. The flexibility advantage of the newer BEs, which are compatible with wider activity windows and/or recognize multiple PAM sequences, is significantly compromised by the high risk of bystander mutations in adjacent nucleotides and off-target effects accompanying these technologies (Li et al., 2022a; Zuo et al., 2019; Lebek et al., 2023). In addition, base editing is currently not applicable for generating insertions or deletions. Delivery of BEs imposes another significant limitation, especially when targeting complex and heterogeneous organs such as the heart. Delivery challenges and advances are thoroughly discussed below.

## Base editing applications in cardiac research

Base editing has been applied in several contexts to study heart disease, from arrhythmias (Wu et al., 2022) to genetic cardiomyopathies (Fig. 2A). Researchers recently described the use of an ABE to target and revert a pathogenic mutation in *LMNA* (c.1824 C>T; p.G608G) in fibroblasts isolated from children diagnosed with Hutchinson-Gilford progeria syndrome (Koblan et al., 2021). Heart failure preceded by premature atherosclerosis, loss of vascular smooth muscle cells and vascular stiffening is the primary cause of death in children with progeria, who have an average lifespan of ∼14 years (Prakash et al., 2018). ABE-mediated genome editing of patient-derived fibroblasts achieved ∼90% genomic DNA correction, as estimated by the assessment of on-target editing through sequencing analysis of total sequencing reads with A-T converted to G-C. Efficient editing led to reduced progerin levels in the corrected cells and restored nuclei to their normal shape (Koblan et al., 2021). Importantly, these results were corroborated in transgenic mice homozygous for the same pathological *LMNA* mutation. In this context, 3-day-old and 2-week-old mice received a single systemic injection [postnatal day (P)3, retro-orbital; P14, retro-orbital or intraperitoneal] of an AAV9 carrying the ABEmax intended to target various progeria-relevant tissues, including heart and muscle. The mice injected at 2 weeks of age exhibited the highest DNA correction rates in multiple targeted tissues at 6 months postinjection. Sequencing analysis revealed ∼30% corrected DNA in the heart and ∼20% in the aorta. In line with the *in vitro* findings, *in vivo* ABE-mediated correction of the pathogenic human *LMNA* c.1824 C>T allele in mice led to a reduction of progerin RNA and protein levels in several clinically relevant tissues. Importantly, correction of the LMNA mutation also translated into an improved vascular function, resulting in an extended lifespan of the treated mice (Koblan et al., 2021). Olson and co-workers have successfully used base editing in a homozygous mouse model of DCM that carries a mutation in the gene encoding RBM20. The *Rbm20*^R636Q^ mutation corresponds to the human pathogenic mutation *RBM20*^R634Q^ due to differences in the number of amino acids in RBM20 of the two species. The homozygous mutant mice recapitulated phenotypic traits observed in human DCM patients, including reduced fractional shortening, atrial and ventricular dilation, heart failure and premature death (Nishiyama et al., 2022). The researchers administered the ABEmax components by intraperitoneal injection of AAV9 in homozygous 5-day-old mice, as cardiac dysfunction was already evident at this age. Evaluation of ABE gene editing in heart tissues revealed 66% of *Rbm20* cDNA transcripts to be precisely corrected. Further functional analysis of the treated mutant mice at 4 and 8 weeks after ABE injection demonstrated substantial cardiac function improvement, as estimated by the >2-fold fractional shortening in treated mice compared to mutant non-treated mice. In parallel, cardiac chamber size was partially restored to normal dimensions, whereas cardiac dilation was rescued in the treated mice at the age of 12 weeks (Nishiyama et al., 2022).

Base editing has also been used to correct a pathogenic mutation in the *Myh6* gene (*Myh6*c.1211C>T) that is associated with HCM. In this study, the ABEmax-NG system was used to correct the mutation in zygotes and embryos (*in utero* correction) of *Myh6*-mutant HCM mice, achieving an editing efficiency of between 62.5% and 70.8% of the treated embryos. The correction reduced the expression levels of mutant RNA and rescued the HCM phenotype in postnatal mice and their offspring (Ma et al., 2021). Two recent studies shed more light on the therapeutic potential of base editing in the context of HCM. Researchers from the Olson and from the Seidman laboratories published two independent studies focusing on the *in vivo* correction of the pathogenic HCM-linked dominant-negative c.1208G>A (p.R403Q) variant in β-myosin (*MYH7*) (Chai et al., 2023; Reichart et al., 2023). In one of the studies, the authors used hiPSCs to screen various ABEs, opting for an editor with a narrow editing window. The relatively low editing efficiency of the chosen editor (close to 34% of corrected reads) was balanced by the lower risk bystander edits. Treatment of patient-derived hiPSC-derived cardiomyocytes heterozygous for the c.1208G>A mutation with the selected ABE resulted in functional improvement as assessed by reduction of contractile force generation and restoration of ATP consumption to normal levels. Next, the researchers generated a humanized mouse model carrying the same β-myosin pathogenic mutation in a homozygous and a heterozygous manner. Heterozygous mice presented with HCM-related symptoms, such as ventricular hypertrophy, myocyte disarray and fibrosis, by the age of 9 months. Because the *MYH7* c.1208G>A mutation only exists in a heterozygous form in human patients, the researchers performed intrathoracic injections of a dual AAV9-ABE system to correct the pathogenic mutation in cardiomyocytes in newborn heterozygous pups. In addition, mice received a special diet high in 0.1% cyclosporin to accelerate HCM onset and progression. Editing efficiency was estimated to be at ∼32% of the target pathogenic adenine in cardiomyocytes, without bystander editing. Compared with the untreated mice, the mice that received the ABE injection showed reduced traits of HCM, such as wall thickening, and had similar echocardiographic read-outs to those of wild-type mice (Chai et al., 2023). The other study, Richter et al. (2020), assessed both an ABE and a CRISPR-Cas9 approach to correct or silence, respectively, the c.1208G>A pathogenic mutation. For the ABE approach, the researchers injected mouse models harboring the pathogenic variant intrathoracically at the age of 10-13 days. Mice received a dual AAV9-Abe8, a particularly active deaminase (Richter et al., 2020), which resulted in an editing efficiency of more than 70% of the ventricular cardiomyocytes. Treatment prevented the onset of HCM cardiac pathology and dysfunction for 32 weeks, although bystander edits were detected at a rate of 3-5% together with a low, but significant, rate of off-target editing. In parallel, the investigators employed the classical CRISPR-Cas9 approach to introduce indels in the target gene and eventually silence the c.1208G>A mutation in cardiomyocytes. Intrathoracic injection of a single AAV9-CRISPR-Cas9 vector prevented HCM onset in mouse models. However, high-dose treatment was linked to defective cardiac function from editing the wild-type allele, pointing to a narrow therapeutic window.

As well as directly correcting disease-causing mutations, base editing has evolved as a great tool for inducing exon skipping (Box 2). A recent example of this is the use of ABEmax to correct the DMD-causing mutation, *DMD* ΔEx51. This approach produced a local editing efficiency of 35% in myofibers and restored dystrophin in the ΔEx51 mice (Chemello et al., 2021). Genome editing-mediated restoration of dystrophin levels through the exon skipping approach was also possible in patient-specific hiPSC-derived cardiomyocytes, which constitutes a more clinically relevant target for the prevention of DMD-associated DCM (Sabatine et al., 2017; Dick et al., 2013). Importantly, exon-deletion mutations accumulate in a specific region of the *DMD* gene and are responsible for 68% of all total mutations, with the deletion of exon 51 being the second most frequent genetic alteration. This suggests that targeting ΔEx51 could potentially be of therapeutic value for a large cohort of DMD patients (Chemello et al., 2021). An interesting approach developed to potentially treat ischemic heart disease was conceptualized by Lebek et al. (2023), who, using base editing, were able to remove the oxidation activation sites of CaMKIIδ, which is a major contributor to cardiac disease. Their experiments conducted on hiPSC-derived cardiomyocytes under hypoxic conditions demonstrated that editing the CaMKIIδ (*Camk2d*) gene to remove methionine residues that are sensitive to oxidation provides protection against ischemia/reperfusion (IR) injury. In addition, by editing the CaMKIIδ gene in mice during IR, they observed that it promoted recovery of heart function that would have otherwise been severely damaged. Therefore, the investigators suggested that gene editing of CaMKIIδ has the potential to serve as an advanced and permanent therapy for heart disease (Lebek et al., 2023). BE components have also been delivered to the liver using lipid nanoparticles (LNPs; which are discussed further in later sections of this Review), resulting in the efficient knockout of proprotein convertase PCSK9 in healthy cynomolgus monkeys (Rothgangl et al., 2021; Musunuru et al., 2021). *PCSK9* is highly associated with cardiovascular disease, as the protein it encodes plays a key role in regulating blood cholesterol levels by controlling the number of low-density lipoprotein (LDL) receptors on the surface of liver cells. LDL receptors are responsible for removing LDL from the bloodstream, and PCSK9 works by binding to and promoting the degradation of these receptors, thus reducing their availability and increasing blood cholesterol levels. Previous studies have shown that individuals with mutations that enhance PCSK9 levels have elevated LDL cholesterol, and are at increased risk of developing cardiovascular disease, including heart attacks and strokes. Conversely, individuals with mutations that result in low levels of PCSK9 have lower LDL and lower risk of cardiovascular disease (Abifadel et al., 2003; Cohen et al., 2006; Sabatine et al., 2017). Both Rothgangl et al. (2021) and Musunuru et al. (2021) reported that using BEs to knock out *PCSK9* in the monkeys successfully reduced their blood cholesterol levels, with no substantial off-target editing or severe immune response. *PCSK9* targeting is supposed to be advantageous as it constitutes a one-off, permanent and precise approach to potentially overcoming the side effects of the currently used oral LDL inhibitors, such as statins (Adhyaru and Jacobson, 2018). These studies have paved the way for developing BE-mediated knockout into a therapy that can be applied in the clinic after it has been sufficiently optimized for use in humans.

The above-mentioned studies have given valuable evidence that base editing came to the cardiac field to stay. However, we should not neglect specific limitations accompanying BE applications. For example, in multiple studies, high editing efficiency *in vivo* was achieved by using a high AAV dose (Chemello et al., 2021; Koblan et al., 2021; Lebek et al., 2023). More specifically, Chemello et al. (2021) reported that they injected 12-day-old mice with a viral dose theoretically equivalent to the systemic dose of 1.5×10^16^ vector genomes (vg)/kg, with the highest AAV9 dose permitted by the U.S. Food and Drug Administration (FDA) being 3×10^14^ vg/kg. The safety and efficacy of these treatments need to be evaluated in larger animals and at lower vector doses to avoid viral toxicity-related side effects and immunogenicity. Additionally, a deeper analysis of potential off-target editing and interaction with other drugs is missing from most of the currently available studies, regardless of the low off-target effects assessed at the most likely predicted sites. Furthermore, the *RBM20* study conducted by Nishiyama et al. (2022) used a mouse model that is homozygous for the pathogenic mutation, whereas human patients carry heterozygous RBM20 mutations, and homozygous patients show premature death. To increase clinical relevance, the authors suggest the conduction of long-term studies of heterozygous mice and investigations into right ventricular dysfunction before and after the correction (Nishiyama et al., 2022). Overall, despite challenges and safety concerns, base editing has the potential to revolutionize the treatment of genetic disorders of the heart and other organs in the future.

## Prime editing technology

Prime editors (PEs) are HNH nuclease-inactivated nCas9 proteins that are fused to engineered reverse transcriptase (RT) domains and directed to target sites by engineered sgRNAs, known as prime editing guide RNAs (pegRNA) (Fig. 2A) (Anzalone et al., 2019). pegRNAs encode the target 5′ protospacer sequence, the scaffold sequence, and an extension at the 3′ end that contains a primer-binding sequence (PBS), the desired edit and a region of homology with the genomic DNA. On binding to the desired target site, the Cas9 RuvC nickase domain nicks the PAM-containing DNA strand, facilitating hybridization of the liberated 3′ end of the genomic target site with the PBS of the pegRNA extension. Successful hybridization primes reverse transcription using the pegRNA extension as a template. The newly synthesized (c)DNA strand exists as a 3′ DNA flap that contains the newly desired edit, which competes to replace the original unedited 5′ DNA flap. The permanent installation of the edit occurs after DNA repair, which uses the new 3′ DNA flap as a template (Anzalone et al., 2020).

Extensive research has been conducted to optimize PE and pegRNA structure due to the original system's considerable variability in editing efficiency among target sites and cell types. Currently, the most promising PE systems are the PEmax editors (Box 2) combined with engineered pegRNAs (epegs; Box 2), which are currently considered the gold standard (Chen et al., 2021b; Nelson et al., 2022; Doman et al., 2022; Chen and Liu, 2022). The ability of the pegRNAs’ PBS and RT template (RTT) to encode a wide variety of sequences highlights this system's versatility. Simultaneously, predicting editing efficiency remains the biggest limitation (Kim et al., 2021; Mathis et al., 2023; Koeppel et al., 2023). In addition, reverse transcription into the pegRNA scaffold has been reported; however, incorporation into the genome has typically been low (Anzalone et al., 2019).

## Prime editing’s potential in cardiac research

Prime editing is of particular interest for modeling and correcting mutations associated with cardiac disease because it can target a wide range of single-nucleotide variants and more complicated genetic alterations such as short deletions or insertions (Fig. 2B,C) (Anzalone et al., 2019). Multiple studies have successfully used prime editing in hiPSC cultures (Surun et al., 2020; Chemello et al., 2021; Chen et al., 2021b). Among these, Chemello et al. (2021) used prime editing to restore DMD protein levels in hiPSC-derived cardiomyocytes carrying the *DMD* ΔEx51 mutation. The strategy restored the correct open reading frame of the mutated *DMD* to produce wild-type DMD. This subsequently rescued the arrhythmic defect observed in the mutant cardiomyocytes, as evidenced by the restored arrhythmic calcium traces upon editing (Chemello et al., 2021). Despite the high efficiency of base editing of the DCM-associated p.R634Q mutation in the *RBM20* gene (Nishiyama et al., 2022), base editing cannot correct all *RBM20* mutations as it is limited by a narrow activity window, undesired editing and the lack of a proper PAM sequence near some target nucleotides. To overcome these limitations, Nishiyama et al. (2022) used PE to correct the p.R636S (c.1906C>A) mutation, which is also associated with DCM. The PE3b max combined with epegRNA led to a correction rate of 40% of mutant transcripts in an hiPSC line harboring the homozygous p.R636S mutation. Deep sequencing analysis showed no detectable mutations at any of the potential off-target sites (Nishiyama et al., 2022).

Interestingly, a study from Li et al. (2022b) has shown that prime editing is, overall, a more efficient and precise technique for introducing heterozygous point mutations in induced pluripotent stem cells (iPSCs) compared to CRISPR-Cas9 and transcription activator-like effector nuclease (TALEN) editing technologies. Indeed, prime editing can achieve ∼60% efficiency, especially when combined with editors delivered as mRNAs and transcribed *in vitro* (Li et al., 2022b). However, prime editing's *in vivo* applications are currently restricted to treating liver disease, largely because current delivery methods preferentially target hepatocytes rather than cardiac or other tissue (Liu et al., 2021a; Zhi et al., 2022; Bock et al., 2022).

Prime editing is a highly valuable tool that holds great promise for the treatment of cardiac disease. Its editing flexibility and ability to correct indels and introduce large deletions, which base editing cannot achieve, make it a highly effective method. Although relatively new to the cardiac research field, prime editing has already demonstrated efficiency in generating cell and animal models of DMD (Zhou et al., 2022c; Happi Mbakam et al., 2022a,b; Chemello et al., 2021), which could eventually help identify new drug targets or potential therapies for cardiac disease. Compared to other gene editing technologies, PE has the advantage of reduced off-target effects, making it a highly desirable therapeutic option. However, its application to treat cardiac conditions has been significantly limited by the low efficiency of heart cell genome editing *in vivo*. Although the studies we discuss here have demonstrated the potential of prime editing, improved editing and delivery tools are needed to bring it closer to the bedside. The necessary improvements are discussed in the following sections of this article.

## Efficiently delivering gene editing tools to the heart

Efficient delivery of gene editing components is crucial for the success of genetic editing, especially in the context of therapies. Editing techniques rely on the precise delivery of components such as sgRNAs and nucleases to target cells to induce precise changes in the genome. Effective delivery methods ensure that the editing components reach the target cells and penetrate the cell membrane, avoiding degradation by the immune system or other cellular defense mechanisms to reach the nucleus intact. By achieving efficient delivery, gene editing can be targeted and specific, minimizing off-target effects and maximizing the therapeutic benefits of gene therapy. Thus, optimizing delivery methods is an essential step towards developing safe and effective gene therapies. Although the delivery of gene editing components has been successfully developed and tested in various disease models *in vivo*, the specific targeting of cardiomyocytes remains a challenge (Bock et al., 2022; Carreras et al., 2019; Chemello et al., 2021; Chen et al., 2021a; Davis et al., 2022; Finn et al., 2018; Georgiadis et al., 2021; Zhou et al., 2022b). In this section, we overview the main systems that facilitate the delivery of PEs and BEs to the heart.

### AAV as a vehicle for gene editing machinery

AAVs are popular viral vectors that are used to deliver gene therapy agents in various disease models *in vivo*, in clinical trials and in clinically approved therapies (Ferrari et al., 2021; Gillmore et al., 2021; Wang et al., 2020a; Dunbar et al., 2018; Keeler and Flotte, 2019). Among the naturally occurring serotypes, AAV serotypes 1, 5, 6, 8 and 9 have shown tropism (Box 2) to the heart, with a higher preference for skeletal muscle cells than for cardiomyocytes (Liu and Olson, 2022; Li and Samulski, 2020). To prevent a gene editor from being expressed in non-cardiac tissue, researchers often use cardiac-specific promoters to drive the expression of the editor (Qiu et al., 2022; Nishiyama et al., 2022). Recently, new variants of AAV9 have been developed to generate more cardiomyocyte-specific delivery systems. The AAV9 variant ‘AAVMYO’ shows increased specificity to efficiently transduce skeletal and cardiac muscle compared to peripheral tissues in mice *in vivo*, which was discovered after a directed evolution approach to identify functional capsid variants with tissue specificities in mice *in vivo* (Weinmann et al., 2020). ‘MyoAAV’ was similarly developed by directed evolution in mice and in non-human primates to generate a muscle-tropic capsid for *in vivo* gene delivery (Tabebordbar et al., 2021). The therapeutic potential of these AAV variants is highlighted by their cardiac specificity and by the low dose required to achieve efficient transduction of gene editors to desired cell types, thus reducing dose-limiting toxicity (Liu and Olson, 2022; Wilson and Flotte, 2020).

**Fig. 3.**
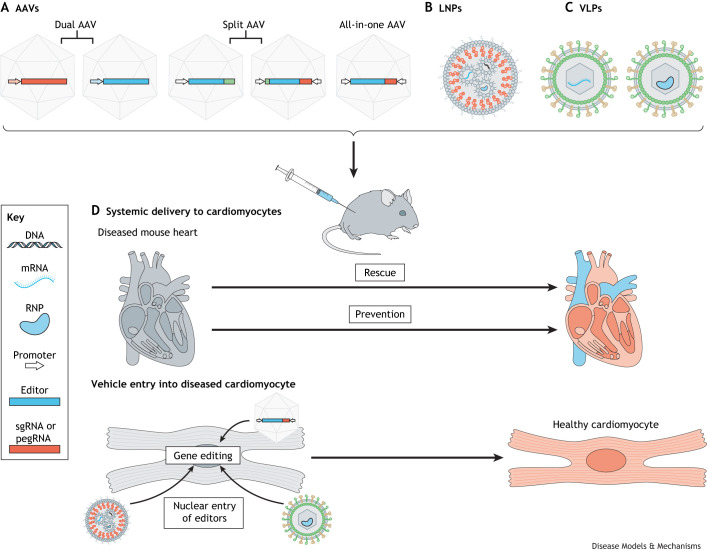
**Schematic presentation of vehicles that can be used to deliver gene editing agents to the heart *in vitro and in vivo*.** (A) AAVs are often used to deliver gene editing agents to the heart *in vitro* and *in vivo*. Various strategies have been developed, of which the dual AAVs, the split AAVs and the all-in-one AAVs have been most successful. In dual-AAV strategies, the sgRNA and Cas9 are delivered separately in two distinct viral particles. In split-AAV strategies, gene editors are divided in an N-terminal and C-terminal half in two separate AAVs. Once the cargo is co-transduced in the same cell, recombination can be accomplished at DNA, mRNA or protein level. Non-canonical compacter Cas variants have been developed and used in all-in-one AAV base editing approaches for simultaneous delivery of the sgRNA and editor by the same vector. (B) LNPs are able to deliver gene editing agents as DNA, mRNA or protein. Diverse LNP compositions have been developed containing variable amounts of PEG lipids, cholesterol, structural lipids and ionizable lipids. (C) VLPs have been used to carry editing agents as mRNA and protein to desired tissues *in vivo*. (D) AAVs, LNPs and VLPs can be intravenously injected to study the result of gene editing in the context of (genetic) heart disease. After release of the cargo to the desired cells, the gene editor machinery will transfer to the nucleus to complete incorporation of the desired edit. AAV, adeno-associated virus; LNP, lipid nanoparticle; pegRNA, prime editing guide RNA; RNP, ribonucleoprotein; sgRNA, single-guide RNA; VLP, virus-like particle.

Numerous studies have utilized AAVs as vehicles for *in vivo* gene therapy in mouse models for cardiomyopathies such as ACM, HCM and DCM (Li et al., 2021a; Yadav et al., 2019; Wang et al., 2019, 2020b; Liu et al., 2021b; Dave et al., 2022). However, the maximum length of the transgene cassette that can be inserted into AAVs, at ∼4.7 kb, precludes them being used to carry canonical BEs and PEs for gene therapy (Raguram et al., 2022; Wang et al., 2019, 2020a; Newby and Liu, 2021; Liu and Olson, 2022). To overcome such limitations, ‘dual AAV’, ‘all-in-one AAV’ and ‘split AAV’ systems have been developed (Fig. 3A). In dual-AAV strategies, the sgRNA and Cas9 are delivered separately using two distinct viral particles. A challenge when using dual AAV is to simultaneously deliver sgRNA and Cas9 to the desired tissue, which is important as editing efficiency greatly depends on sgRNA abundance (Min et al., 2019; Hakim et al., 2018). To circumvent the separate delivery of sgRNA, all-in-one AAV base editing approaches have been developed that use non-canonical compact Cas variants (Fig. 3A). However, these non-canonical Cas variants require the recognition of more complex and less frequent PAM sequences, leading to increased off-targets and are often unapplicable for genomic sites of interest (Davis et al., 2022; Chen et al., 2022; Zhang et al., 2021a, 2022; Kim et al., 2022; Anzalone et al., 2020). In split-AAV strategies, gene editors are divided into N-terminal and C-terminal halves in two separate AAVs (Fig. 3A). Once the cargo is co-transduced into the same cell, DNA, mRNA or protein recombination can take place (Fig. 3D) (Wang et al., 2020a; Ryu et al., 2018). Split-AAV strategies are designed using a split-intein system (Box 2) approach, which currently provides the most promising tool for efficiently delivering BEs and PEs *in vivo* and has been used in various disease models studying (metabolic) liver, eye, inner ear, neurological, skeletal muscle and developmental disease (Choi et al., 2022; Xu et al., 2021; Ryu et al., 2018; Villiger et al., 2021, 2018; Zhou et al., 2022b; Rothgangl et al., 2021; Carreras et al., 2019; Chadwick et al., 2017, 2018; Zheng et al., 2022; Gao et al., 2022; Li et al., 2021b; Koblan et al., 2021; Bose et al., 2021; Levy et al., 2020; Lim et al., 2020; Yang et al., 2020; Liu et al., 2022a; Yeh et al., 2020; Hong et al., 2022; Arbab et al., 2023). Most recently, these split-intein systems were applied *in vivo* to prevent the onset of genetic cardiomyopathies in HCM and DCM mouse models. In two promising studies, both studying the human pathogenic variant R403Q in *MYH7*, researchers were able to prevent the development of HCM in humanized mouse models by injection of split-AAV9 serotypes carrying ABEs (Chai et al., 2023; Reichart et al., 2023). In a preprint from Grosch and co-authors, the recently described AAVMYO capsid was applied to treat P635L and R636Q *Rbm20* mutation-induced DCM in mice, using a split-intein derived ABE (Grosch et al., 2022 preprint). The treatment significantly improved the molecular and physiological defect associated with *Rbm20* DCM. Interestingly, the researchers treated 4-week-old mice with smaller doses of viral particle compared to Nishiyama and colleagues’ treatment of 5-day-old mice (Nishiyama et al., 2022), showing the tremendous potential of optimized tissue-specific viral capsids.

Although the usage of AAVMYO and MyoAAV is still emerging, the original AAV9 system can efficiently transduce murine cardiomyocytes *in vivo*, which makes it an attractive tool for studies of (genetic) heart disease. However, optimal gene editing therapy should have limited off-targets and side effects. AAVs are known to be persistently expressed over many years, which is ideal for gene overexpression therapy, but increases the risk of off-targets and bystander mutations in gene editing therapy, especially when CBEs or regular Cas9 are used (Li et al., 2022a; Zhou et al., 2022a; Yu et al., 2020; Lee et al., 2020; Grunewald et al., 2019; Zuo et al., 2019). Self-inactivating CRISPR-AAV systems, which express a sgRNA that targets its own Cas enzyme, have been developed to overcome this problem of prolonged editor expression; however, they could not completely diminish it (Ibraheim et al., 2021; Li et al., 2019). In addition, immune responses against the foreign gene-editing proteins and viral capsids are potentially harmful (Ling et al., 2021). Moreover, the cardiomyocyte-specific promotor may be sufficient to retain gene editor expression to cardiomyocytes, but it does not prevent viral transduction in other tissues. The viral entry into other cell types could therefore still lead to harmful side effects (Baldrick et al., 2023).

### LNPs as vehicles for gene editing machinery

LNPs have emerged as a key and increasingly popular system for drug delivery, particularly since they received FDA approval to intravenously deliver liver small interfering RNAs (siRNAs) to inhibit the synthesis of transthyretin in patients with hereditary transthyretin-mediated amyloidosis (Akinc et al., 2019) and to intramuscularly administer severe acute respiratory syndrome coronavirus 2 (SARS-CoV-2) mRNA vaccines (Baden et al., 2021; Polack et al., 2020; Paunovska et al., 2022). LNPs contain four main components to encapsulate RNAs, proteins or ribonucleoproteins (RNPs): (1) a structural lipid forming the fundamental scaffold; (2) cholesterol for stabilization and membrane fluidity, elasticity and permeability; (3) cationic or ionizable lipids to load negatively charged nucleic acids; and (4) stealth lipids to evade or reduce immunogenicity (Fig. 3B) (Paunovska et al., 2022; Raguram et al., 2022; Rohner et al., 2022; Hou et al., 2021; Rurik et al., 2022; Altinoglu et al., 2015; Zhang et al., 2021b). LNPs naturally tend to accumulate in the liver because of their APOE-induced hepatocyte uptake (Akinc et al., 2019; Paunovska et al., 2022). Hence, most scientists have used LNPs to deliver *in vivo* gene editors to the livers of animal models (Fig. 3D) (Gillmore et al., 2021; Musunuru et al., 2021; Akinc et al., 2019).

LNPs that carry mRNA-encoded Cas9 were first systemically administered to a humanized mouse model of hereditary tyrosinemia, corrected only 0.8% of the liver and generating indels in 24% of liver cells (Yin et al., 2016). In follow-up studies, LNPs with mRNA-encoded Cas9 successfully knocked out *Pcsk9* and transthyretin (Yin et al., 2017; Finn et al., 2018). More recently, LNPs have successfully delivered mRNA- and RNP-encoded BEs to the livers of mice and non-human primates (Musunuru et al., 2021; Villiger et al., 2018; Jiang et al., 2020; Rothgangl et al., 2021; Zhang et al., 2020a; Packer et al., 2022; Song et al., 2020). For example, Villiger and co-authors were able to reduce l-phenylalanine, an amino acid that phenylketonuria patients cannot metabolize, below the therapeutic threshold of 360 μmol/l in a phenylketonuria (*Pah^enu2^*) mouse model treated with an LNP-packaged BE (Villiger et al., 2021). In addition, disruption of *PCSK9* by LNP-mediated delivery of BEs reduced LDL to therapeutically relevant levels, demonstrating a treatment option for atherosclerotic cardiovascular disease in mice and non-human primates *in vivo* (Musunuru et al., 2021; Rothgangl et al., 2021). Both studies reported minimal off-targeting at the DNA and RNA levels, which could be explained by the transient expression of mRNA-encoded gene editors packaged in the LNPs compared to long-term expression of AAV-delivered gene editors (Villiger et al., 2021). However, only a small number of hepatocytes could be assessed using whole-genome sequencing in these quality control experiments; hence, a hepatocyte population with increased off-targets could be missed. The long-term effect of gene editor expression in the heart has yet to be studied, as has the effect of increased off-target edits. A recent report showed that, during ageing, cardiomyocytes accumulate single-nucleotide variants at a faster rate than many dividing cells. This study showed distinctive mutational signatures in cardiomyocytes that implicated failed nucleotide and base excision repair and defective mismatch DNA repair, leading to widespread oxidative genotoxicity (Choudhury et al., 2022). One could speculate that long-term expression of gene editors carries detrimental effects and increased risk for off-target edits, highlighting the importance for further research.

Despite the promising preclinical results in *in vivo* models of liver disease, the use of LNP to deliver gene editors to other organs remains a challenge due to their preferential hepatocyte uptake. Local injections of lipid-encapsulated mRNA and RNP gene editors to the mouse inner ear and retina have been successful (Yeh et al., 2018; Zuris et al., 2015; Gao et al., 2018; Jang et al., 2021). However, local injections often cannot be used, particularly when treating heart disease. One solution to this is to administer multiple doses of gene editors. Swanck and co-workers improved editing efficiencies from 10% to 21% in whole-liver lysates from *Pah*-mutant mice by re-dosing of mRNA-encoded CBEs carried by LNPs injected in the tail vein (Villiger et al., 2021). Repeated intravenous dosing of LNPs carrying mRNA-encoded BEs or PEs that are only transiently expressed might thus be a feasible approach for treating genetic heart disease and might overcome the inefficient targeting of cardiomyocytes. In addition, the prolonged expression of gene editors after AAV-mediated delivery and the increased risk of off-targets make LNPs an attractive vehicle to overcome these problems.

LNPs have also been engineered to target specific cell types, which efficiently transfer Cas9 or its variants to the liver, brain, lung and muscle (Wei et al., 2020a,b). Such strategies include altering their lipid composition and conjugating tissue-specific receptors (Cheng et al., 2020; Paunovska et al., 2022; Dilliard et al., 2021; Godbout and Tremblay, 2022). For example, Scalzo et al. (2022) tested multiple mixtures of LNP formulations to successfully transfect cardiomyocytes *in vitro*, the best formulation of which subsequently successfully delivered plasmid DNA encoding GFP to the murine heart *in vivo* upon tail vein injection. To our knowledge, cardiomyocyte-specific targeting has not yet been achieved. Another strategy, conjugating an anti-T-cell-receptor antibody to LNPs that carry mRNA to generate therapeutically relevant chimeric antigen receptor T (CAR-T) cells *in vivo*, was successful in a mouse heart failure model. Treated mice generated CAR-T cells and eliminated fibroblast activation, which decreased interstitial fibrosis and increased left ventricular function (Rurik et al., 2022). Engineered LNPs that target cardiomyocyte-specific receptors could potentially lead to higher gene editing efficiency in genetic heart disease, but this remains to be demonstrated.

### Virus-like particles as vehicle for gene editing machinery

Virus-like particles (VLPs) are derived from existing viral scaffolds and exploit their natural properties. They lack potentially harmful viral genetic material but enable efficient intracellular delivery of cargo (Lyu et al., 2020; Raguram et al., 2022; Zdanowicz and Chroboczek, 2016; Ikwuagwu and Tullman-Ercek, 2022). Immature retroviruses are often used to generate VLPs because of their large particle diameter (100-200 nm) and ability to provide sufficient space to package Cas9, PEs or BEs as mRNA, protein or RNP (Fig. 3C) (Zhang et al., 2015; Banskota et al., 2022; Choi et al., 2016; Koblan et al., 2021; Lyu et al., 2021). VLPs take advantage of the tropisms and efficiency of a retroviral capsid while avoiding viral or cargo integration into the host genome. As such, VLP-delivered mRNAs, proteins and RNPs are only transiently expressed, limiting the potential for harmful long-term effects, as have been described for AAV-mediated BE delivery. Initial *in vitro* studies targeting primary T cells and microglia with VLPs produced only modest knockout efficiencies (14-28%) using CRISPR-Cas9; however, substantial improvements (70-90% editing efficiency) have since been achieved by VLP-delivered gene editors in HEK293T and immune cells (Campbell et al., 2019; Choi et al., 2016; Hamilton et al., 2021; Lyu et al., 2019; Mangeot et al., 2019; Indikova and Indik, 2020). To date, only a handful of *in vivo* studies have successfully VLP-delivered gene editors as RNPs to the mouse liver and eye, achieving knockout efficiencies of up to 44% (Mangeot et al., 2019; Ling et al., 2021). Engineered VLPs (eVLPs) substantially increase on-target gene editing following their intravenous administration into mouse models of eye and liver disease (Fig. 3D) (Banskota et al., 2022). These results were comparable to those of AAV- and LNP-delivered BEs; however, minimal editing efficiency (<4%) was shown in the heart (Banskota et al., 2022). Nevertheless, the low targeting efficiency of VLPs to the heart could be overcome by generating eVLPs from cardiotropic viruses (Sahoo et al., 2021). To our knowledge, a successful VLP-mediated gene editor delivery system that can target the heart has yet to be developed.

## Recent improvements to genome editing efficiency

Recent research efforts have focused on optimizing delivery systems for BEs and PEs and on increasing their efficiency in specific organs and cell types. Alongside this work, efforts are ongoing to optimize editing technologies further to increase their flexibility and to eliminate off-target and other undesired effects. Recently, Rosello et al. (2022) described an advanced BE that is no longer restricted to the PAM-dependent window. Through extensive testing of multiple CBEs in zebrafish, these authors identified CBE4max-SpRY, which had been previously reported as an almost PAM-less BE that recognizes nearly every PAM sequence in cultured cells (Walton et al., 2020). They then used this CBE4max-SpRY system to simultaneously base edit multiple genes in zebrafish to model melanoma predisposition (Rosello et al., 2022). To further broaden the editing flexibility of BEs, researchers have also developed the dual BE, A&C-BEmax, which contains a Cas9 fused to both A and C deaminases (Zhang et al., 2020b). This new tool allows both C-to-T and A-to-G conversions at the same target site with substantially fewer off-target effects. Moreover, new PE tools that use dual pegRNAs, known as TwinPEs, have recently been reported. The extensions of these dual pegRNAs are complementary to each other and each one targets a different DNA strand. These new tools increase PE precision and facilitate the correction or complementation of large or complex pathogenic alleles (Anzalone et al., 2022). In addition, a recent study developed drive-and-process arrays to facilitate multiplex base editing and prime editing in human cells (Yuan and Gao, 2022). These arrays contain tandem-assembled transfer RNA (tRNA)-guide RNA (gRNA) arrays, with the individual gRNAs being released by the endogenous tRNA processing machinery. These platforms are likely to become valuable for studying polygenic and complex genetic diseases in the future. To increase safety, a different study performed by Cui et al. (2022) described the use of irreversible small molecules, known as selective inhibitors of nuclear export (SINEs), to inhibit Cas9 in the context of genome, base or prime editing tools. More precisely, SINEs, which include the FDA-approved anticancer drug KPT330, can halt Cas9 activity by interfering with the nuclear export process of Cas9 mRNA. This approach aims to eliminate the off-target effects caused by a continuously active Cas9. Several studies have also introduced the use of anti-CRISPRs (Acrs) to develop more controllable and precise CRISPR editing tools (Marino et al., 2020). Acrs are naturally produced phage proteins used as a resistance mechanism against the bacterial CRISPR-Cas system, which bacteria produce in response to bacteriophage infection (Marino et al., 2020). Similarly to Acrs, Ades are deaminases produced by viruses to defend themselves against the antiviral activity of the bacterial APOBEC3 (Uriu et al., 2021). Of note, a recent study described four virus-derived deaminases as being more efficient CBE-inactivating tools than Acrs (Liu et al., 2022b). Acrs or Ades hold promise for future therapeutic applications, as they offer the possibility to control the timing and duration of CRISPR activity, thus enhancing safety of the gene editing therapeutic approaches.

## Future perspectives

CRISPR, an ancient tool to protect bacteria from phage infections, has now become a vitally important tool for molecular research. Strikingly, BEs have already entered clinical trials for sickle cell disease (NCT05456880), β-thalassemia (NCT05442346) and familial hypercholesterolemia (NCT05398029). For sickle cell disease and β-thalassemia, the treatment relies on *ex-vivo* electroporation followed by autologous transplantation of the edited cells, while the familial hypercholesterolemia trial uses LNP-encapsulated BEs intravenously delivered to turn off the *PCSK* gene in the liver. From a broader perspective, gene editing therapeutics hold the promise of providing a single, definitive treatment offering lasting benefits, alleviating patients from the obligation to adhere to prolonged non-specific medical regimens that may negatively impact their quality of life. In the context of cardiomyopathy, this is a particularly pertinent issue, as existing treatment strategies are primarily centered around attenuating disease symptoms, for example through interventions such as antiarrhythmic drugs, catheter ablation, use of implantable cardioverter defibrillators, as opposed to directly curing the disease. Undoubtedly, the incorporation of gene therapy approaches into clinical practice is trusted to yield significant impacts.

In November 2022, Verve Therapeutics, the developer of hepatocyte *PCSK9* BE in familial hypercholesterolemia patients, was informed by the FDA that the program has been placed on hold due to safety concerns regarding the BE. Three main challenges needed to be addressed: (1) understanding the variations in editing potency between human and non-human cells, (2) mitigating the risks associated with germline editing, and (3) conducting thorough off-target analyses in cell types beyond hepatocytes. Although there is currently no good argument to suggest that the FDA’s hold on the Verve trial will not be lifted, this recent development highlights the critical importance of addressing and understanding the outstanding questions surrounding safety and possible patient implications of genome editing. Open communication and collaboration can facilitate the sharing of resources and expertise, and help accelerate the development and translation of genome editing therapies for cardiomyopathy. Prime editing has emerged as a promising technique for precise gene editing. However, its efficiency *in vivo* is currently low. A concerted effort to improve the properties of PEs and delivery vectors is necessary to increase the chances of success. Efficient targeting of specific cardiac cell subtypes, such as cardiomyocytes, cardiac fibroblasts, endothelial cells and macrophages, could be greatly improved by identifying unique cell-specific membrane markers and incorporating them into delivery vectors for transporting editing components to the target cell population. This approach would help limit off-target effects. Furthermore, the development of tools to regulate the timing and duration of CRISPR activity will be crucial in enhancing safety by reducing off-target effects and bystander mutations. Additionally, a more extensive use of larger mammalian models, such as pigs, dogs or non-human primates, could provide valuable information about the dose-specific toxicity and long-term side effects of gene editing therapies for cardiomyopathies. Performance of clinical trials is pivotal for the determination of the optimal dose but also of the right time to intervene, i.e. before or after the onset of disease symptoms. Finally, addressing certain ethical considerations, including safety, informed consent, equity and transparency, is necessary to ensure responsible use of genome editing therapeutic strategies (Almeida and Ranisch, 2022).

Overall, precision genome editing holds great promise for treating and potentially curing genetic heart disease, given the latest developments and the incredible speed at which the field is progressing. Deepening our understanding of the safety profile of these technologies will be crucial to fully unlock their therapeutic potential for cardiomyopathies and other cardiac conditions.
